# Physiological responses to retinopathy of prematurity screening: indirect ophthalmoscopy versus ultra-widefield retinal imaging

**DOI:** 10.1038/s41390-025-03906-4

**Published:** 2025-02-13

**Authors:** Ravi Purohit, Fatima Usman, Amanda Ie, Marianne van der Vaart, Shellie Robinson, Miranda Buckle, Luke Baxter, Michelle Clee, Amanda Clifford, Eleri Adams, Rebeccah Slater, Chetan K. Patel, Caroline Hartley, Kanmin Xue

**Affiliations:** 1 Oxford Eye Hospital, Oxford University Hospitals NHS Foundation Trust, Oxford, UK; 2 Department of Paediatrics, University of Oxford, Oxford, UK; 3 Newborn Care Unit, Oxford University Hospitals NHS Foundation Trust, Oxford, UK; 4 Great Ormond Street Hospital for Children NHS Foundation Trust, London, UK; 5 Nuffield Laboratory of Ophthalmology, Nuffield Department of Clinical Neurosciences, University of Oxford, Oxford, UK

## Abstract

**Background/Aims:**

Retinopathy of prematurity (ROP) screening is vital for early disease detection in very premature infants but can cause physiological instability. This study compares the physiological response to binocular indirect ophthalmoscopy (BIO) with indentation and non-contact ultra-widefield (UWF) retinal imaging in non-ventilated neonates. The impact of the Dandle WRAP, a specialised swaddling aid, on UWF imaging was also assessed.

**Methods:**

This retrospective study included 86 ROP screening events in 66 non-ventilated infants aged 35.3 weeks (range 30.6–44.6). Vital signs were continuously recorded, evaluating immediate (within 15 min) and longer-term (within 12 h) physiological responses.

**Results:**

ROP screening significantly increased heart and respiratory rates and decreased oxygen saturation within 15 min of screening. No significant differences in physiological responses were found between BIO and UWF imaging, although there was a trend towards lower maximum heart rate with UWF imaging. The Dandle WRAP did not significantly alter physiological responses but improved the ease and speed of UWF imaging.

**Conclusion:**

UWF imaging does not increase physiological instability compared to BIO in non-ventilated infants. Specialised swaddling aids may facilitate the imaging procedure.

**Impact:**

- ROP screening can be distressing for premature infants and induce physiological instability during and after the examination. We deployed non-contact ultra-widefield retinal imaging as the default method of ROP screening and show that it induces comparable physiological responses as traditional indirect ophthalmoscopy in non-ventilated babies. Dandle WRAP swaddling facilitated handling and speed of retinal imaging. The study demonstrates that imaging-based ROP screening is safe and efficacious in non-ventilated neonates, and continuous multimodal physiological recordings can provide detailed assessment of the effects of procedures and medications.

## Introduction

**Fig. 1:**
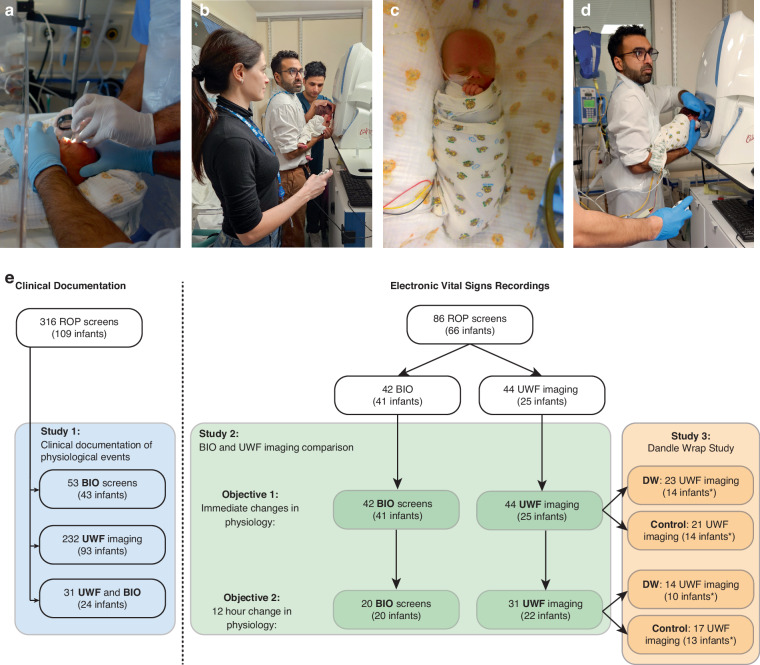
Screening methods and study flowchart. **a**–**d** Illustrative examples of ROP screening techniques and use of the Dandle WRAP. Parents provided written consent for the use of images. **a** An infant undergoing traditional ROP screening by binocular indirect ophthalmoscopy (BIO) with indentation. **b** An unswaddled infant undergoing ultrawidefield (UWF) retinal imaging in the ‘flying baby’ holding position. Note that the infant’s arms needed to be stabilised by an assistant while a second assistant captured the images. **c** An infant fully swaddled with a Dandle WRAP whilst resting in their incubator. **d** An infant swaddled with a Dandle WRAP undergoing UWF imaging. Note that the legs can be removed from the Dandle Wrap to improve stability and safety in the flying baby position. As the infant’s arms are securely swaddled, only one assistant was needed to capture the images. **e** Study flowchart. For Study 3, infants were divided into groups according to whether they were swaddled using the Dandle WRAP (DW) or unswaddled (control group) during UWF imaging. Note that infants in the control group were swaddled with a conventional blanket whilst in their cot/incubator but were not swaddled during UWF imaging as it would be unsafe to perform the flying baby hold with a full swaddle.

Retinopathy of prematurity (ROP) is a major preventable cause of childhood blindness, affecting ~30% of premature infants born <32 weeks gestation.^1^ ROP screening enables early disease detection and treatment, which is crucial for management and preventing complications including sight loss. Traditional ROP screening involves binocular indirect ophthalmoscopy (BIO) with scleral indentation to visualise vascularisation up to the ora serrata, which requires a high level of technical expertise^2,3^ (Fig. 1a). Moreover, despite use of topical anaesthesia and comfort measures (such as swaddling), infants often demonstrate significant signs of distress and physiological instability, such as tachycardia, elevated blood pressure, desaturation and apnoea.^4–7^ To date, no pain reduction strategies have been shown to be completely effective for ROP screening.^8,9^

Alternative screening strategies may improve physiological stability. We have previously demonstrated the utility of 200-degree non-contact ultra-widefield (UWF) scanning laser ophthalmoscopy for ROP assessment, with the infant being lifted out of the cot/incubator and held to the camera in the ‘flying baby’ position^10^ (Fig. 1b). UWF imaging allows consistent visualisation of the posterior pole and peripheral retina in a single image, and enables objective monitoring and improved communication between ROP screening and treatment centres.^11^ Since February 2021, UWF imaging has been adopted as the default method of ROP screening at our Level 3 Newborn Care Unit. A previous randomised crossover trial compared physiological responses between UWF imaging and BIO-based ROP screening, assessing several intermittent timepoints up to 10 min after the screening. The results indicated no significant difference between the two methods in terms of changes in heart rate and respiratory rate, but oxygen saturation was significantly lower (by mean 0.8%) during UWF imaging.^12^ While intermittent capture of vital signs is valuable, recent studies have suggested that continuous electronic capture of vital signs enables more detailed evaluation of physiological stability.^13–18^ Moreover, comparison of physiological instability between BIO and UWF imaging over a longer time period is warranted due to increased likelihood of apnoea within 24 h following ROP examination.^4,5^

Swaddling is a widely used non-pharmacological pain management technique for procedural pain in infants.^19,20^ A recent refinement is the Dandle WRAP (Dandle Lion Medical, Danbury, Connecticut), which is made of stretchy fabric and secured using Velcro (Fig. 1c). Its design is particularly useful for performing UWF imaging of infants held in the flying baby position (Fig. 1d). Moreover, it enables safe swaddling of the infant to potentially improve comfort.

Herein, we compare the physiological responses to UWF imaging versus BIO-based ROP screening. We first evaluated the incidences of episodes of physiological instability (including desaturation, apnoea, tachycardia, bradycardia) in a large cohort of infants documented within a clinical database (Study 1) (Fig. 1e). Next, we analysed continuous electronic recordings of vital signs which permit detailed analysis of physiological responses to both screening methods (Study 2). We then investigated using the Dandle WRAP for UWF imaging-based ROP screening (Study 3). Finally, we conducted a survey to determine staff experience with using the Dandle WRAP.

## Methods

### Study design and participants

All infants underwent ROP screening at Oxford Newborn Care Unit, Oxford University Hospitals NHS Foundation Trust, Oxford, UK and were included across three studies (Fig. 1e).

#### Study 1: Comparison of clinically significant events between BIO and UWF imaging

We retrospectively reviewed the clinical notes of all infants who underwent ROP screening over a period of one year from 1 Feb 2021. Screens were excluded if carried out in the outpatient eye clinic. The study was registered as a Trust clinical audit (ref 9353).

#### Study 2: Comparing physiological stability between BIO and UWF imaging using electronic vital signs recordings

Participants were identified from our research database and included if they had vital signs recorded during ROP screening between 2016 and 2022. Note that for Studies 2 and 3 we included data from our research database, which is separate from our clinical database (used for Study 1). Vital signs are not electronically recorded as standard in our unit and so only infants who were enroled in research related to ROP screening during this 7-year period could be included.

UWF imaging using mobile Optomap California platform (Optos, Dunfermline, UK) was adopted as the default method for ROP screening in Jan 2021. The study therefore compared data collected before 2021 (BIO group) with data collected in 2022 (UWF imaging group). Further details on study design are provided in Supplementary Methods, including information on ROP screening. All infants received mydriatic eye drops, topical anaesthetic drops and had a neonatal speculum inserted, irrespective of the ROP screening method.

#### Study 3: Effect of swaddling with Dandle WRAP on physiological stability during UWF imaging

In Sep 2022, the neonatal unit introduced a guideline change advising use of Dandle WRAP for ROP screening. Infants were swaddled from approximately one hour before (at the time of dilating drops) until two hours afterwards. The time at which the WRAP was removed was at the discretion of the nurse. During image capture, the infant’s legs were unwrapped (with upper body swaddled only) to allow the legs to straddle the clinician’s forearm while being held in the flying baby position (Fig. 1d). After completion of imaging and return to cot, total body swaddling with legs placed back in the Dandle WRAP was provided. All infants screened with UWF imaging (included in Study 2) were also included in Study 3 and split according to whether they were swaddled with a Dandle WRAP (Dandle WRAP group) or not swaddled (control group) (Fig. 1e). In addition, we conducted a staff survey to assess opinions and practice of using the Dandle WRAP (Supplementary Methods), which was registered as a Trust service evaluation (ref 8476).

### Data collection

#### Documentation of cardiorespiratory events from clinical notes (Study 1)

Vital signs during ROP screening were monitored by a neonatal nurse and recorded in case notes using established event definitions. Desaturation was defined as oxygen saturation <80% for ≥10 s; tachycardia as heart rate >200 bpm for ≥15 s; bradycardia as heart rate <100 bpm for ≥15 sec; apnoea as a respiratory pause >20 s.^21^

#### Vital signs recordings (Study 2 and 3)

All infants included in Studies 2 and 3 had their heart rate, respiratory rate, and oxygen saturation monitored using Philips IntelliVue MX800/750 clinical monitors, which is also part of standard care. The data was continuously downloaded to a laptop using ixTrend software (ixitos GmbH, Germany). The start and end of ROP examination was marked by a researcher pressing a key on the recording laptop. The start of the ROP screen was annotated prior to the clinical team handling the baby (i.e., before instilling local anaesthetic eye drops, or any handling for positioning of the head; and in the case of UWF imaging, before infants being lifted from the cot). The end of the ROP screen was annotated after both eyes had been screened and the speculum removed; and in the case of screening using UWF imaging, when the infant was also placed back in their cot/incubator. If more than one ophthalmologist screened the infant, the end of the ROP screen was when all screening had finished. Note that different ophthalmologists screened the infants.

### Data analysis

Data analysis code is available on the Paediatric Neuroimaging Group Gitlab (https://gitlab.com/paediatric_neuroimaging/rop-vital-signs.git). The individual infant physiological responses to ROP screening, along with screening method and basic demographic information are provided in the Supplementary Material.

#### Analysis of clinically documented cardiorespiratory events (Study 1)

Data were collated in FileMaker Pro (Claris International Inc., California) and exported to Microsoft Excel (Microsoft Corp, Washington). Summary metrics (frequency of tachycardia, bradycardia, desaturations, apnoea) were calculated according to the screening method.

#### Analysis of electronic vital signs recordings (Studies 2 and 3)

Analysis was performed using MATLAB (R2021a, MathWorks). Heart rate and oxygen saturation were taken directly from the monitor. Inter-breath intervals were used to calculate the respiratory rate and identify apnoeas using algorithm described by Adjei et al.^13^ (Supplementary Methods).

To calculate immediate physiological response to ROP screening, data was epoched from 5 min before the start of ROP screen until 15 min afterwards. This time window was chosen as the duration of the screen ranged from 1.5 to 10.6 min (average 4.6 min) and visual inspection of the data suggested that immediate physiological response returned to baseline levels within this time window (Supplementary Fig. 2). For each infant, we calculated the maximum and average increase in heart rate and respiratory rate, and minimum and average decrease in oxygen saturation. We also considered longer term changes in physiology, comparing the 12 h following screening with the 12 h before screening, using the same definitions as for Study 1 to identify episodes of physiological instability.^21^

### Statistical analysis

Statistical analysis was performed in Stata and SPSS (v.28.0.0.0). As some infants were studied on multiple occasions, linear mixed effects models were used with participant ID included as a random factor with random intercept and slope. We tested ten outcome variables of physiological response: (i) average heart rate increase, (ii) maximum heart rate, (iii) average oxygen saturation decrease, (iv) minimum oxygen saturation, (v) average respiratory rate increase, (vi) maximum respiratory rate, (vii) number of tachycardias, (viii) number of bradycardias, (ix) number of oxygen desaturations, (x) number of apnoeas; each outcome was tested separately. To quantify physiological response to ROP screening, the mean response was compared for significant differences from zero, adjusting for baseline mean of the corresponding physiological variable (e.g. heart rate in the 5 min or 12 h beforehand). To compare the physiological responses between screening and swaddling methods, linear mixed effects models were used with physiological responses included as dependent variable and screening/swaddling method as independent variable. Gestational age (GA), birthweight (BW), postmenstrual age (PMA), sex, weight, ventilation method at examination and baseline mean of the corresponding physiological variable were included in the models to adjust for any effect. Standardised differences were calculated to assess imbalance between groups (Supplementary Methods).^22^ To adjust for multiple testing, we performed Bonferroni correction using the effective number of tests. This accounts for correlations across the tests, rather than assuming independence of the tests as with standard Bonferroni correction.^23^ We allocated a total alpha of 0.05 to the 10 tests/outcome variables, and estimated the effective number of tests using the method outlined by Li et al.^23^. There were 7.59 effective tests, resulting in a significance threshold of 0.0066. All *p*-values are reported unadjusted, and significance judged relative to this 0.0066 threshold.

## Results

### Study 1: Clinical viability of UWF imaging-based ROP screening

Over a period of one year from Feb 2021, a total of 109 babies underwent ROP examinations on 316 occasions and were included in the retrospective analysis of clinical notes. Of these screening examinations, 232 utilised UWF imaging only, 53 underwent BIO examination only, and 31 used a combination of both methods (Table 1). BIO examinations were performed for the purpose of (i) gold standard discharge from ROP screening, (ii) when significant ROP changes were observed on UWF images, or (iii) when the baby could not be lifted out of the cot (e.g. ventilated infants). Overall, the demographic profile between these groups were comparable (Table 1). On two occasions, UWF imaging was abandoned due to significant apnoea or desaturation. For most infants, UWF imaging was well tolerated, and the frequency of cardiorespiratory events were comparable with BIO examination (Table 1). Respondents to our staff survey showed no clear preference to the screening method (Supplementary Table 3).

**Table 1:** Demographic information and physiological instability among infants in Study 1 (retrospective clinical audit).

	UWF imaging only	BIO only	UWF imaging + BIO
Demographics			
Gestational age at birth (mean [SD], weeks)	26.1 [2.2]	26.4 [2.2]	25.8 [2.2]
Postmenstrual age at exam (mean [SD], weeks)	35.8 [3.6]	36.1 [4.1]	36.9 [3.9]
Birthweight (mean [SD], g)	873 [311]	932 [329]	790 [299]
Weight at exam (mean [SD], g)	1884 [707]	1902 [740]	2429 [668]
ROP grading			
No. of examinations	232	53	31
Stage 0 (no ROP)	55	38	5
Stage 1	21	2	1
Stage 2	29 (17% with popcorn)	3 (0% with popcorn)	9 (33% with popcorn)
Stage 3	48 (62% regressing stage 3)	6 (1.67% regressing stage 3)	12 (17% regressing stage 3)
Stage 4A	0	0	0
Stage 4B	0	0	1
Vascular front not seen	75	0	3
Hazy view	4	3	0
Pre-plus	30	4	10
Plus	11	4	7
Episodes of physiological Instability			
Frequency of tachycardia	12.7% (31/229)	10.5% (4/38)	23% (7/30)
Frequency of bradycardia	0.4% (1/229)	2.6% (1/38)	0% (0/30)
Frequency of desaturation	3.9 (9/229)	7.9% (3/38)	2.1% (1/30)
Frequency of apnoea	0.4% (1/229)	2.6% (1/38)	3% (0/30)

Further details regarding ROP grading in this cohort are given in the Supplementary Results.

### Study 2: Comparison of physiological stability between UWF imaging and BIO ROP screening

In study 2, we compared the physiological response to ROP screening between BIO and UWF imaging in infants whose vital signs were continuously recorded. A total of 86 ROP screening episodes were included, with 44 examinations performed using UWF imaging and 42 examinations performed using BIO (Fig. 1e). Infant demographic information is given in Table 2.

**Table 2:** Demographics of infants included in Study 2 comparing physiological responses to binocular indirect ophthalmoscopy (BIO) versus UWF imaging ROP examinations, and Study 3 comparing infants swaddled with a Dandle WRAP and control infants.

	BIO (*n* = 42)	UWF imaging (*n* = 44)	Standardised difference	Dandle WRAP (*n* = 23)	Control (*n* = 21)	Standardised difference
Gestational age (mean [SD], weeks)	27.8 [2.3]	27.0 [2.5]	0.44	27.3 [2.7]	26.6 [2.2]	0.42
Birthweight (mean [SD], g)	1090 [362]	877 [327]	0.87	921 [337]	830 [316]	0.40
Male sex (%)	21 (50%)	27 (61%)	0.19	15 (65%)	11 (55%)	0.13
Postmenstrual age at examination (mean [SD], weeks)	35.3 [2.4]	35.4 [3.1]	0.28	36.2 [2.9]	34.5 [3.0]	0.82
Weight at examination (mean [SD], g)	2136 [520]	1764 [565]	1.00	1929 [579]	1571 [510]	0.90
Mode of ventilation at examination:						
• Self-ventilating on air	20 (48%)	7 (16%)	0.58	3 (15%)	3 (13%)	0.13
• Low-flow oxygen	8 (19%)	9 (20%)	0.03	4 (20%)	5 (22%)	0.06
• High-flow oxygen	14 (33%)	28 (64%)	0.52	13 (65%)	15 (65%)	0.06
Duration of ROP examination (mean [SD], min)	4.06 [1.82]	5.02 [2.04]	2.05	4.83 [2.12]	5.24 [1.98]	0.29

Infants underwent either (1) BIO (*n* = 42) or (2) UWF imaging (*n* = 44) based ROP screening with continuous physiological monitoring. For Study 3, infants who were screened using UWF imaging were compared according to whether they swaddled with a Dandle WRAP or not. Standardised differences were calculated between the two groups for Study 2 and 3 separately. Larger values indicating larger differences between groups. Standardised differences larger than 0.1 for prognostic variables is viewed as a difference in the demographics between the two groups that requires adjusting in the analysis. Note that duration of ROP examination is not a prognostic variable and so was not adjusted for. The start of the ROP screen was annotated prior to the clinical team handling the baby (i.e. before instilling local anaesthetic eye drops, or any handling for positioning of the head). The end of the ROP screen was annotated after both eyes had been screened and the speculum removed; and in the case of screening using UWF imaging, when the infant was also placed back in their cot/incubator. Duration of ROP screening for BIO group is from the administration of topical anaesthetic drops until the point when the speculum is removed from the second eye. For the UWF imaging group, duration is from the administration of topical anaesthetic drops to when the infant is returned to their cot (after removal of speculum), and consequently includes the time for lifting the infant from their cot).

**Fig. 2:**
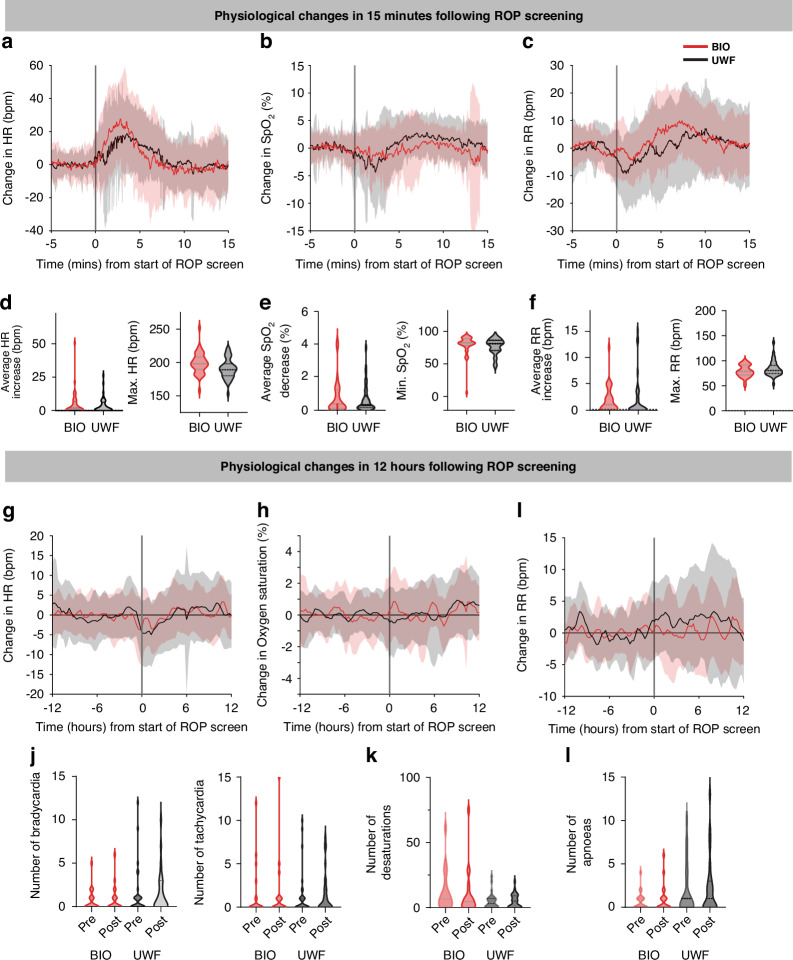
Comparison of physiological changes following ROP screening by UWF imaging versus BIO. **a**–**f** Changes in the 15 min following the start of ROP screening. Changes in **a** heart rate (HR), **b** oxygen saturation (SpO_2_) and **c** respiratory rate (RR) in response to ROP screening. Solid lines indicate the group mean and shaded areas represent standard deviation. Red represents the infants who were screened using Binocular indirect ophthalmoscopy (BIO). Black represents the infants who were screened using UWF (ultra-widefield) imaging. Individual infant traces are baseline corrected by subtracting their pre-procedure mean. Grey vertical line indicates the start of ROP screening. **d**–**f** Comparison of physiological metrics between BIO and UWF imaging groups measured in the 15 min following the start of ROP screening shown using violin plots where the width of the plot represents the number of infants at that value. Horizontal dotted lines indicate mean and interquartile range. **d** Metrics (average and maximum) used to assess HR changes, in beats per min. **e** Metrics used to assess SpO_2_ changes. **f** Metrics used to assess RR in breaths per min. **g**–**l** Changes in the 12 h following the screening. **j** Number of bradycardia (<100 bpm for at least 15 s) and number of tachycardia (>200 bpm for at least 15 s), **k** number of desaturations (<80% for at least 10 s), and **l** number of apnoeas (pauses in breathing of at least 20 s) in the 12 h pre and post screening.

In the first 15 min following the start of ROP screening, there was a significant increase in heart rate and respiratory rate, and a significant decrease in oxygen saturation in both BIO and UWF imaging groups (Fig. 2, Supplementary Table 1). Whilst on average there was an increase in heart rate following the start of ROP examination, some infants experienced bradycardia (Supplementary Fig. 2). There were no significant differences in physiology between the two screening methods (Supplementary Table 1), however, there was a trend towards a lower maximum heart rate in the UWF imaging group (*p* = 0.036, significance level: α = 0.0066 after correcting for multiple comparisons, linear mixed effects model, Fig. 2d, Supplementary Table 1). In the 12 h following the ROP screening, there were no significant differences in the number of bradycardias, tachycardias, oxygen desaturations or apnoeas between the two screening methods (Fig. 2g–l, Supplementary Table 1).

### Study 3: Dandle WRAP facilitates retinal image acquisition but does not significantly alter physiological response

**Fig. 3:**
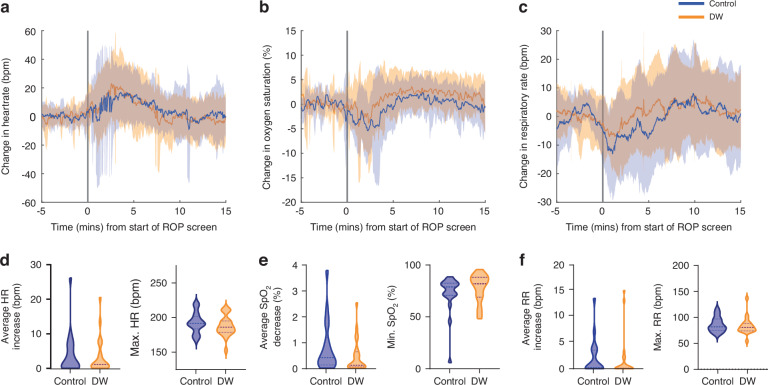
Comparison of infant physiology in response to ROP screening with different swaddling interventions. Changes in **a** heart rate, **b** oxygen saturation and **c** respiratory rate in response to ROP screening. Solid line indicates the group mean and shaded areas the standard deviation. Blue represents Control group infants, orange represents infants who were swaddled using the dandle WRAP (DW). Individual infant traces are baseline corrected by subtracting their pre-procedure mean. Grey vertical line indicates the start of ROP screening. All infants were screening using UWF imaging. **d**–**f** Comparison of physiological metrics between the two groups measured in the 15 min following the start of ROP screening shown using violin plots where the width of the plot represents the number of infants at that value. Horizontal dotted lines indicate mean and interquartile range.

We investigated whether changes in physiology were reduced when infants were swaddled with the Dandle WRAP. All infants in this analysis were screened using UWF imaging. Infant demographic information is given in Table 2. In the 15 min or 12 h following the start of the ROP screen, there was no significant difference in physiology between the Dandle WRAP and control groups (Fig. 3, Supplementary Table 2). However, there was a trend towards a lower maximum heart rate in the Dandle WRAP group (*p* = 0.043, significance level: α = 0.0066, Fig. 3d, Supplementary Table 2).

Our survey of clinical staff showed that most (*n* = 21/22, 95.4%) (Supplementary Fig. 1) respondents preferred using the Dandle WRAP compared with swaddling with a muslin. Moreover, most of the respondents believed the Dandle WRAP was easy to use (*n* = 21/22, 95.4%) and felt babies were very or somewhat comfortable in it (*n* = 22/22, 100%) (Supplementary Figs. 3 and  4).

## Discussion

We present detailed continuous physiological responses (up to 12 h) to ROP screening using two alternative methods – standard BIO examination and UWF retinal imaging. We compared the overall impact of these two screening methods on vital signs changes in non-ventilated neonates. With either method, ROP screening had a significant effect on physiological stability, causing an immediate increase in heart rate and respiratory rate, and decrease in oxygen saturation.

BIO has been the gold standard method for ROP screening. Consequently, current ROP guidelines were formulated based on BIO findings, including the concept of Zones I, II and III.^2^ However, retinal imaging provides the advantage of objective documentation of disease progression with nuanced interpretation such as number/extent of popcorn lesions, and images acquired in local centres can be reviewed remotely by specialised ophthalmologists. Our unit has pioneered the use of UWF imaging in infants, which allows non-contact 200° visualisation of the retina in a single image.^10,11^ Whilst Optos UWF imaging is facilitated by pupil dilation and insertion of a lid speculum, we successfully captured retinal images in poorly dilated or non-dilated pupils in 1% of screening episodes (Supplementary Results). Moreover, compared with contact wide-field imaging (e.g. RetCam 3), the Optos camera does not touch the cornea and avoids the need for scleral depression which has been shown to illicit transient oculocardiac (or trigeminovagal) reflex or even retinal haemorrhages during ROP screening.^24,25^ In this study, we did not detect any overall difference in physiological response between BIO and UWF imaging. However, there was a trend towards lower maximum heart rate in infants screened using UWF imaging. This effect was relatively small but may warrant further investigation in a larger prospective study. By using vital signs monitoring rather than clinical notes or intermittent time points, our results provide a continuous representation of the physiological responses to ROP screening and the basis for future work in this area.

Importantly, infants included in Studies 2 and 3 were either self-ventilating or receiving high-flow or low-flow oxygen. An essential point for clinical teams conducting ROP screening to consider is the suitability of the individual infant to undergo examination. In our clinical audit, two UWF imaging attempts were aborted due to significant apnoea and desaturations. These events can also occur during BIO examination. Thus, whilst overall UWF imaging and BIO give rise to similar physiological responses, individual infants may exhibit exaggerated responses. As UWF imaging is conducted with infants in the ‘flying baby’ position^10^ (Fig. 1b), infants who are very unstable may not respond well to the additional handling. While UWF imaging is more challenging in ventilated infants, we have previously demonstrated this to be technically possible if necessary.^26^

Comfort measures such as swaddling are often recommended to reduce the physiological response to painful procedures in infants.^2,19,20^ During the study period, the clinical care team introduced the Dandle WRAP for ROP screening in our unit. An exploratory sample was used, matching a similar number of infants swaddled with the Dandle WRAP with those studied before the guideline change. While there were no significant differences in mean vital sign parameters between infants swaddled with a Dandle WRAP compared to standard care, use of the Dandle WRAP reduced the examination duration by an average of 25 s. In our experience, the Dandle WRAP is particularly useful for UWF imaging, which is facilitated by swaddling of the arms (Fig. 1d). Whilst non-pharmacological pain relief techniques are insufficient to prevent physiological instability during ROP screening,^8^ this adjunct is simple to implement without risk of adverse effects and improves feasibility of fundus imaging in the flying baby position.

This study was a retrospective analysis and infants were not randomised between screening or swaddling methods. Consequently, there was some imbalance between BIO and UWF imaging groups, e.g. in terms of birth weight and mode of ventilation; and between Dandle WRAP and control groups for PMA (Table 2). Whilst we adjusted for these factors in our statistical analysis, a randomised controlled trial is needed to truly discern these effects. Another limitation results from the BIO and UWF imaging groups coming from two consecutive time periods. In the period when BIO was used, infants who received high flow oxygen therapy (*n* = 14, 33%) would have received oxygen that was manually adjusted by clinical staff, whereas in the latter period when UWF imaging was used, high flow oxygen therapy was automatically adjusted in relation to the infant’s oxygen saturation. Whilst there was no difference in oxygen saturation changes between the two screening methods, this could explain the difference between our study and that reported by Fung et al.^12^ who found that oxygen saturations were 4% lower immediately after completion of UWF imaging than BIO without scleral indentation. However, unlike the work of Fung et al., our data used continuous monitoring of vital signs and are less likely to be affected by sporadic fluctuations from point measurements. Additionally, different ophthalmologists conducted the ROP screening. A prospective study should be conducted and control for these effects.

Interestingly, we noticed that in infants screened using either method, heart rate appears to drop (by 5 bpm) from approximately one hour before the screening until four hours afterwards (Fig. 2g). This likely corresponds to the instillation of mydriatic eye drops 1 h before the scheduled ROP screening and the time for the systemic effects to wear off, and is consistent with previous observations.^27,28^ The continuous quantitative approach demonstrated in this study could be applied to future studies evaluating the adverse effect profiles of different mydriatic drops.^29^

In summary, we found comparable physiological responses to ROP screening in premature infants using BIO and UWF imaging. This study was not designed to compare the efficacy and accuracy of the screening methods. Nevertheless, we show that UWF imaging can be safely performed for routine ROP screening of non-ventilated infants. Imaging enables objective documentation of disease status, tracking of progression, and telemedicine applications. Finally, whilst swaddling with a Dandle WRAP did not significantly alter the physiological response to UWF imaging-based ROP screening, the specialised swaddle helped to facilitate the handling and speed of imaging infants in the flying baby position.

## Supplementary information

Supplementary Materials

Supplementary data

Supplementary data

## Data Availability

The individual infant physiological responses to ROP screening, along with screening method and basic demographic information are provided in the Supplementary Material. Data analysis code is available on the Paediatric Neuroimaging Group Gitlab (https://gitlab.com/paediatric_neuroimaging/rop-vital-signs.git). The raw datasets generated during and/or analysed during the current study are available from the corresponding authors on reasonable request.
