# Supplementary material for: Physiological responses to retinopathy of prematurity screening: indirect ophthalmoscopy versus ultra-widefield retinal imaging
Source: Pediatr Res. 2025 Feb 13;98(5):1801–8. doi: 10.1038/s41390-025-03906-4 (PMC12602345; doi:10.1038/s41390-025-03906-4)
Supplement: Supplementary file 1 — Supplementary Materials [file 41390_2025_3906_MOESM1_ESM.pdf]

SUPPLEMENTARY MATERIAL

Physiological responses to retinopathy of prematurity screening: indirect ophthalmoscopy versus ultra-widefield retinal imaging

Ravi Purohit[1#], Fatima Usman[2#], Amanda Ie[1], Marianne van der Vaart[2], Shellie Robinson[2], Miranda Buckle[2], Luke Baxter[2], Michell Clee[3], Amanda Clifford[3], Eleri Adams[3], Rebeccah Slater[2], Chetan K Patel[1,4], Caroline Hartley[2*], Kanmin Xue[1,4,5*]

[1]Oxford Eye Hospital, Oxford University Hospitals NHS Foundation Trust, Oxford, UK; [2]Department of Paediatrics, University of Oxford, Oxford, UK; [3]Newborn Care Unit, Oxford University Hospitals NHS Foundation Trust, Oxford, UK; [4]Great Ormond Street Hospital for Children NHS Foundation Trust, London, UK; [5]Nuffield Laboratory of Ophthalmology, Nuffield Department of Clinical Neurosciences, University of Oxford, Oxford, UK

[#]These authors contributed equally; *These authors contributed equally

***Corresponding authors**: **Caroline Hartley**, Department of Paediatrics, University of Oxford, Level 2 Children's Hospital, John Radcliffe, Oxford, OX3 9DU, UK. Email: caroline.hartley@paediatrics.ox.ac.uk. **Kanmin Xue**, Nuffield Laboratory of Ophthalmology, Nuffield Department of Clinical Neurosciences, University of Oxford, Level 6 West Wing, John Radcliffe Hospital, Headley Way, Oxford OX3 9DU, UK. Email: kanmin.xue@eye.ox.ac.uk

Supplementary Methods

Study design: Study 2 - Comparing physiological stability between BIO and UWF imaging using electronic vital signs recordings

A total of 86 ROP screening examinations in 66 infants were included: 42 ROP screens in 41 infants were included in the binocular indirect ophthalmoscopy (BIO)-based screening group and 44 ROP screens in 25

infants were included in the UWF imaging screening group. Immediate changes in physiology to the ROP screening in the 15 minutes after the start of the screening (compared with 5 minutes before) were compared between the two groups. In a subset of 42 infants (**Figure 1**) who had their vital signs recorded for at least 12 hours before and after the screening, changes in physiology were also assessed across this longer time period.

Participants were identified from our research database and were included in the current study if they had vital signs recorded during an ROP screen. This included 15 infants recruited for the Procedural Pain in Premature Infants (Poppi) clinical trial[1] and who received a placebo (the trial examined the analgesic efficacy and safety of morphine for procedural pain in premature infants, the infants who received morphine were excluded here), and data from 26 other infants undergoing BIO screening, some of which has been previously published[2]. Data from UWF imaging has not been previously reported.

All infants were born before 32-week gestation or with birth weight <1501 g according to UK guidelines for ROP screening and were included if they were below 42 weeks postmenstrual age (PMA) at the time of the study. Infants were excluded from the study if they were receiving analgesics at the time of the study (aside from topical proxymetacaine drops given immediately before ROP screen), if they were born to mothers who regularly used opiates during pregnancy, or if they were receiving conventional ventilation.

ROP screening techniques

All babies' eyes were dilated using 0.5% cyclopentolate and 2.5% phenylephrine hydrochloride, instilled twice, 5 minutes apart, approximately an hour before the examination. Topical 0.5% proxymetacaine hydrochloride was instilled in both eyes immediately prior to screening.

Binocular indirect (BIO) ROP examination

Each infant was positioned in a supine position in their cot or incubator with their head stabilised by an assistant. After topical 0.5% proxymetacaine was instilled in both eyes, the infant was swaddled and a neonatal speculum (either Acrofine or Malosa) was inserted. Binocular indirect ophthalmoscopy (BIO) was performed with a 28D lens. A neonatal scleral indentor (Acrofine) was used to stabilise the eye and indent

the nasal and temporal ora assisted by a slight head turn in each direction. The speculum was then removed and the examination was repeated for the other eye.

Ultra-widefield (Optomap) imaging-based ROP examination

UWF imaging used an Optomap California (Optos Inc, Dunfermline, UK). After topical 0.5% proxymetacaine was instilled in both eyes, the infant was lifted out of the cot and held in cradle position by the examiner. A neonatal speculum was inserted by the assistant and the cornea was hydrated with a balanced salt solution (BSS). The infant was then transferred to the flying baby position with legs straddling the examiner's dominant forearm and angles of the mandible held by the dominant hand (**Figure 1B, D**). The examiner's non-dominant hand was used to guide the infant's head towards the camera and adjust the level of neck extension. Gentle pressure can be applied to the superior aspect of the speculum to mitigate the Bell's phenomenon during imaging. When the eye is at the optimal distance from the camera aperture, the assistant captured the images. The infant was returned to the cradle position and the speculum moved to the other eye. Following imaging the infant is returned to the cot or incubator. Care is taken throughout the process not to dislodge any cables or lines.

For infants with significant ROP changes seen on the UWF images (i.e. pre-plus or plus disease and/or stage 3 ROP), a BIO screening is performed after imaging. These infants were excluded from the 12-hour analysis of vital signs as they received both screening techniques during the 12 hours.

Vital signs recordings (Study 2 and 3)

Heart rate, (peripheral) oxygen saturation, and pulse (calculated by the monitor) were downloaded continuously onto the external laptop at a sampling rate of 1 Hz, the ECG was downloaded at a sampling rate of 250 Hz from three electrodes placed on the infant's chest, the impedance pneumograph downloaded at a sampling rate of 62.5 Hz from the chest electrodes, and the photoplethysmography at a sampling rate of 125 Hz from a probe placed on the infant's foot.

Analysis of electronic vital signs recordings (Studies 2 and 3)

Heart rate was analysed using the heart rate signal from the monitor, which is derived from the ECG signal, using inbuilt Philips algorithms. In 7 recordings (where ECG was not monitored as the infant was deemed by the clinical care team to be too fragile to have ECG monitoring, or due to technical difficulties with the recording) the pulse from the pulse oximeter was used for heart rate analysis. Oxygen saturation was analysed using the oxygen saturation signal recorded directly from the monitor. The respiratory rate from the monitor was not used in the analysis due to known problems of apnoea calculation in infants[2–4]. Instead, inter-breath intervals (IBI) were identified from the IP using the algorithm described by Adjei et al. 2021[2], which was developed and validated for the identification of IBI and apnoeas in preterm infants. The respiratory rate was calculated in 60-second windows in increments of 1 second as 60 divided by the mean IBI. The respiratory rate was not calculated if more than 50% of the signal in a given window was missing due to artefact[2].

For each infant in the 15 minutes following the ROP screen, we calculated the maximum and average increase in heart rate and respiratory rate, minimum and average decrease in oxygen saturation:

- Maximum heart rate – maximum heart rate within 15 minutes following the start of the screening
- Minimum oxygen saturation – minimum oxygen saturation within 15 minutes following the start of screening
- Maximum respiratory rate – maximum respiratory rate within 15 minutes following the start of screening
- Average heart rate increase – area under the curve within 15 min following the start of screening above a threshold divided by time (i.e. 15 minutes). The threshold was calculated as the infant's mean plus two standard deviations of their 5 min of baseline (**Supplementary Figure 2**). If the HR did not go above the baseline threshold, then the average heart rate increase was 0.
- Average oxygen saturation decrease - the average area above the curve below a threshold of the baseline mean minus two standard deviations.

- Average respiratory rate increase – the average area below the curve above a threshold of the baseline mean plus two standard deviations.

In the 12 hours before and after the screen, we calculated the number of episodes of bradycardia, tachycardia, oxygen desaturation and apnoea. Episodes of apnoea, oxygen desaturation, tachycardia or bradycardia that occurred within 60 sec of the previous episode of that type were counted as single events. To visually compare the heart rate, respiratory rate and oxygen saturation in the 12 hr before and after ROP screening we also calculated the average in 1 hr windows, in increments of 15 min.

Calculation of standardised difference

Standardised differences were calculated according to the approach of Austin[5].

For continuous variables (gestational age, birthweight, postmenstrual age at examination, weight at examination, duration of examination), the standardised difference was calculated as:

$$d = \frac{(\bar{x}_O - \bar{x}_B)}{\sqrt{\frac{s_O^2 - s_B^2}{2}}}$$

Where x̅ denotes the sample mean and s the standard deviation in the UWF imaging group (O) and the BIO group (B).

For categorical variables (sex, mode of ventilation), the standardised difference was calculated as:

$$d = \frac{p_O - p_B}{\sqrt{\frac{p_O(1-p_O) + p_B(1-p_B)}{2}}}$$

Where p denotes the proportion/prevalence within each group.

Staff survey on opinions of the use of Dandle WRAP

An online questionnaire using Microsoft Forms was used to assess staff opinion about the ease of using the Dandle WRAP, whether they preferred the Dandle WRAP to conventional swaddling, and whether they felt infants were comfortable in the Dandle WRAP. We also asked whether they prefer BIO or UWF imaging for ROP screening. The questionnaire consisted of 13 questions, with either multiple-choice Likert-type questions or free-text options. A convenience sample targeting all eligible staff directly involved with ROP screening and /or post-procedure care was used. The questionnaire link was emailed to all the nurses in the Newborn Care Unit and the Ophthalmology team. A QR code was also made available within the unit for participants to scan on their phones and quickly access the survey. Participation was voluntary and results were anonymised.

The questions were as follows (free text questions are indicated, multiple choice options enabled one box to be selected):

1. What is your job role? (Free text)

2. In your opinion, how settled/ comfortable are babies when swaddled with the Dandle WRAP?

[ ] Very comfortable

[ ] Somewhat comfortable

[ ] Neither comfortable nor uncomfortable

[ ] Somewhat uncomfortable

[ ] Very uncomfortable

3. How easy was the wrap to use?

[ ] Very easy

[ ] Easy

[ ] Neither easy nor difficult

[ ] Difficult

[ ] Very difficult

4. Would you prefer to use the Dandle WRAP or swaddling with a muslin?

[ ] Dandle WRAP [ ] Muslin swaddle

Why? (Free text)

5. Would you use the Dandle WRAP regularly if each baby had their own?

[ ] Yes [ ] Sometimes [ ] No

If you answered 'No' or 'Sometimes' to question 5, why not? (Free text)

6. Do you feel there are any drawbacks to using the Dandle WRAP?

[ ] Yes [ ] No

If you answered 'yes', please provide details. (Free text)

7. Please provide any further comments or suggestions you have on the use of Dandle WRAP for ROP screening.

8. Have you ever used the Dandle WRAP during ROP screening?

[ ] Yes [ ] No

If no, why not? (Free text)

9. Have you used the Dandle WRAP for other procedures or comfort care?

[ ] Yes [ ] No

If ‘No’, could you tell us why you haven’t used it?

[ ] Don't know about the Dandle WRAP

[ ] Don't feel confident using the Dandle WRAP

[ ] I don't look after babies needing ROP screen

[ ] I don't think it was suitable for any of the babies

[ ] I haven't been taught how to use the Dandle WRAP

10. Have you been taught how to use the Dandle WRAP?

[ ] Yes [ ] No

11. How confident do you feel about explaining to families why we swaddle or use the Dandle WRAP?

[ ] Very confident

[ ] Confident

[ ] Neither confident nor unconfident

[ ] Unconfident

[ ] Very unconfident

12. In your opinion, which method of ROP screen do you prefer babies to have?

[ ] Optos Screen (Retinal photos using the big camera/ out of bed procedure)

[ ] Indirect screen (using the headset/in-bed procedures)

[ ] No preference

13. Could you tell us why you prefer the ROP method you chose in question 12 or if no preference, why?

(Free text)

Supplementary Results

Details of ROP grading for infants included in Study 1

Between 1 Feb 2021 and 31 Jan 2022, a total of 109 consecutive babies underwent ROP examinations on 316 occasions and were included in the audit of clinical notes. Of these screening examinations, 232 utilised UWF imaging only, 53 used BIO only, and 31 used a combination of both (**Table 1**).

For UWF-based ROP screening examinations, a mean of 6 images (SD = 2.8) were captured per eye across the whole cohort. The mean duration of image capture, defined as the timestamp of the first to last image, was 172 sec (range = 21 - 771 sec, median = 154 sec, IQR = 114-199 sec). Based on the UWF images, ROP grading was performed by consensus by a team of experienced ROP screeners using a set of standardised nomenclature[6,7] (Table 1). Zone 1 was not visible (in its totality) on the captured UWF images in only 4 out of 526 eyes examined, which were subsequently examined using BIO. Plus disease was diagnosed in 5% of imaging-only examinations and pre-plus in 13% of examinations. Regressing plus, based on comparison with the previous imaging examination, was noted in 7% of examinations. The remaining 75% of examinations noted no plus disease. In terms of ROP staging, no ROP stage was diagnosed in 24% of UWF examinations, stage 1 in 9%, stage 2 in 13% (of which 17% was associated with popcorn lesions), and stage 3 in 21% (of which 60% were regressing stage 3). In 32% of imaging examinations, the vascular front was not visualised on any images thus staging was not possible, even though plus disease could be excluded. In 2% of examinations, the vascular front was not visible due to a hazy cornea or tunica vasculosa lentis, however, the posterior pole was visible and therefore plus disease could be ruled out. 1% of eyes were not dilated or did not respond to mydriatic eye drops and were examined without dilatation.

Staff survey on opinions of the use of Dandle WRAP in general

A total of 22 responses were included in the analysis (17 nurses and 5 doctors, approximately 12% of those invited via email, **Supplementary Fig.1**). The majority of participants (n=21/22, 95.5%) had used the Dandle WRAP during ROP screen whilst one participant had used it for patient cares only. Almost all (n=21/22, 95.4%)

respondents had been taught how to use the Dandle WRAP. All but one participant preferred the Dandle WRAP to swaddling with a muslin. Only one respondent preferred a muslin, saying “Both are easy. Muslin is larger for covering baby when compared to Dandle’. Similarly, most participants (n=21/22, 95.4%) said the Dandle WRAP was easy to use (**Supplementary Figure 3A**). Most participants (n=18/22, 81.8%) were also confident about explaining the use of swaddling to families (**Supplementary Figure 3B**).

The majority (n=15/22, 68.2%) of respondents said they would use the Dandle WRAP regularly. By contrast, only 2 (9.1%) participants said they would not use it routinely for cares, with one participant saying Dandle WRAP use would “depend on the infant’s condition” while the other felt “it would affect their neural development”. Just under a quarter (n=5/22, 22.7%) of the respondents said they would only ‘sometimes’ use the Dandle WRAP and the reasons given were:

“Some babies don’t seem to settle with wrap and some bigger babies get quite hot”.

“I think babies need to move freely in a cot”.

“Depends on the babies’ condition”.

“It doesn’t prove useful”.

“I don’t often attend the ROP round anymore”.

Whilst 14/22 (63.6%) had no general comments about the use of Dandle WRAP, 8/22 (36.4%) provided feedback saying:

“The material is really good, and it's helpful most of the time”.

“Very effective!”

“Love them!”

“I have only used it a couple of times but did think it worked very well”.

Respondents offered the following comments/ recommendations regarding the use of the Dandle WRAP:

“We need more supply”.

“It’s a bit tricky on babies with a cannula on the foot”.

“I find them easy to use, but occasionally, struggle to find one to fit a bigger baby”.

“I like to use it. But it's less in number”.

Staff preference between UWF imaging and BIO based technique

Half (n=11/22, 50%) of the participants preferred the BIO method of ROP screen, while about one-third (n=8/22, 36.4%) chose UWF imaging and less than a quarter (n=3/22, 13.6%) had no preference. Supplementary Table 3 shows the reasons reported by the respondents for their ROP screening method preference.

Supplementary Figures

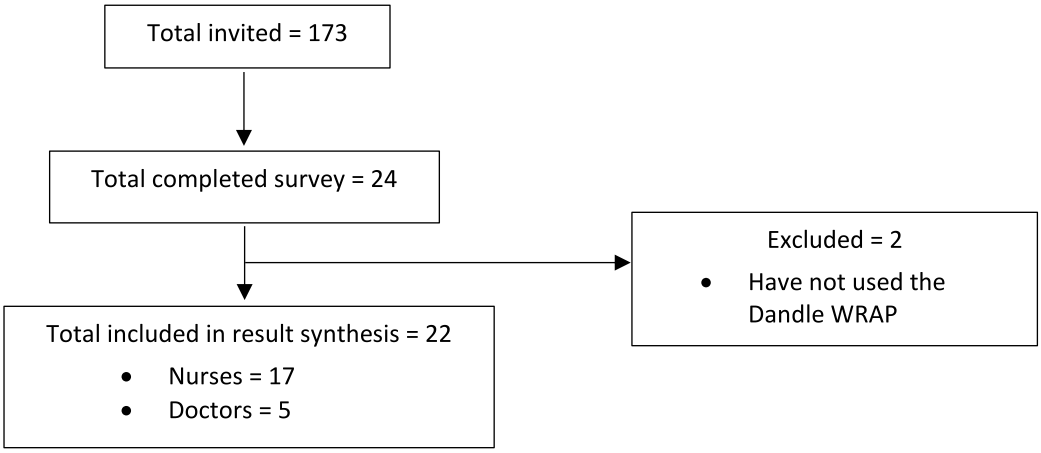

Supplementary Figure 1: Flowchart of responders for the Staff Survey on opinions of Dandle WRAP. Relevant staff (neonatal nurses and the ophthalmology team) were contacted via email.

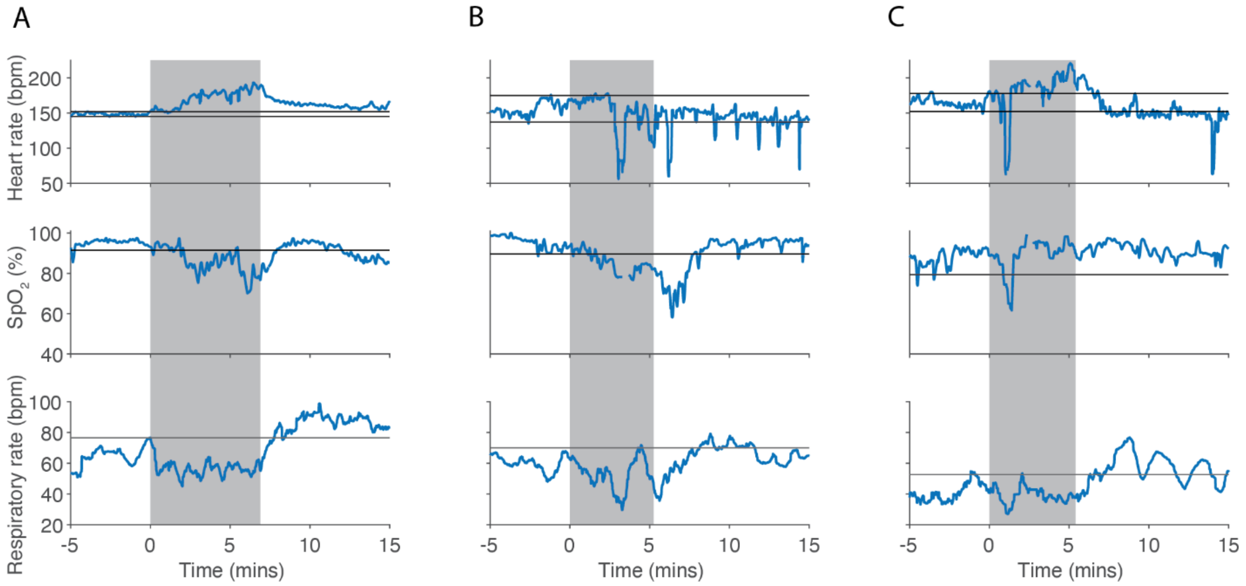

Supplementary Figure 2: Example vital signs traces in individual infants. Heart rate (in beats per minute), oxygen saturation (SpO2) and respiratory rate (in breaths per minute) in three example infants for 5 minutes before the ROP screen until 15 minutes afterwards. Time = 0 is the start of the ROP screen, the grey shaded area indicates the duration of the screen. Horizontal black lines indicate the infants' mean plus/minus the standard deviation of their 5-minute baseline (pre-ROP screen) values. (A) An infant whose heart rate increases and oxygen saturation decreases during the examination. Their respiratory rate increases after the examination. (B) This infant has multiple bradycardias during and

after the examination and their oxygen saturation also drops significantly. (C) The third infant has an immediate bradycardia at the start of screening followed by an increase in heart rate. After an immediate drop in oxygen saturation, their oxygen saturation then recovers and remains within the baseline range for the rest of the 15 minutes.

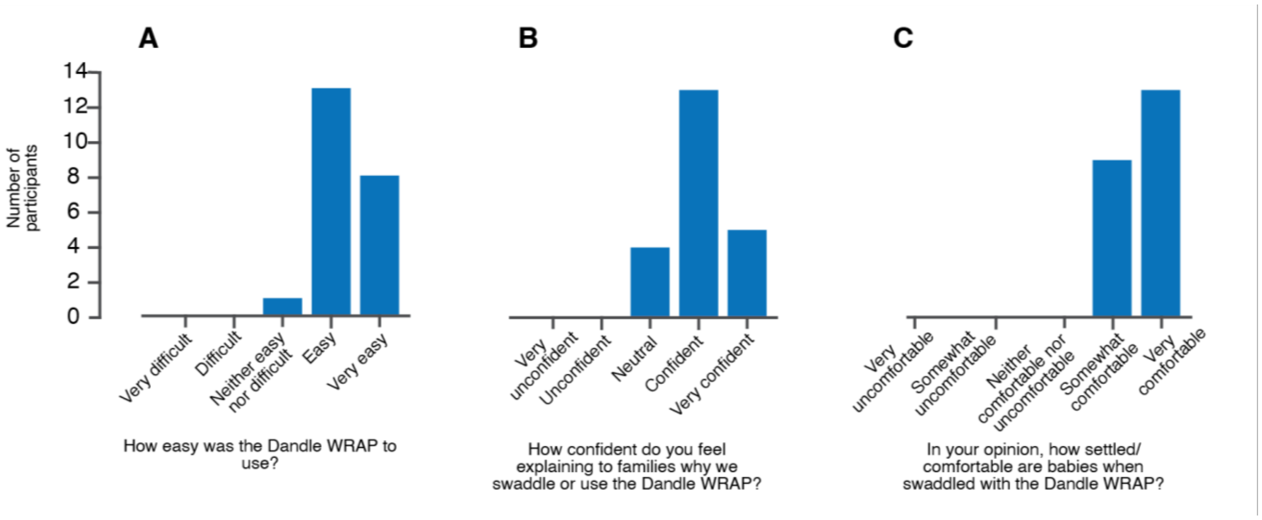

Supplementary Figure 3: Staff opinion on the use of Dandle WRAP. Number of participants who responded with given responses to the three multiple choice survey questions exploring staff opinion of Dandle WRAP use in general.

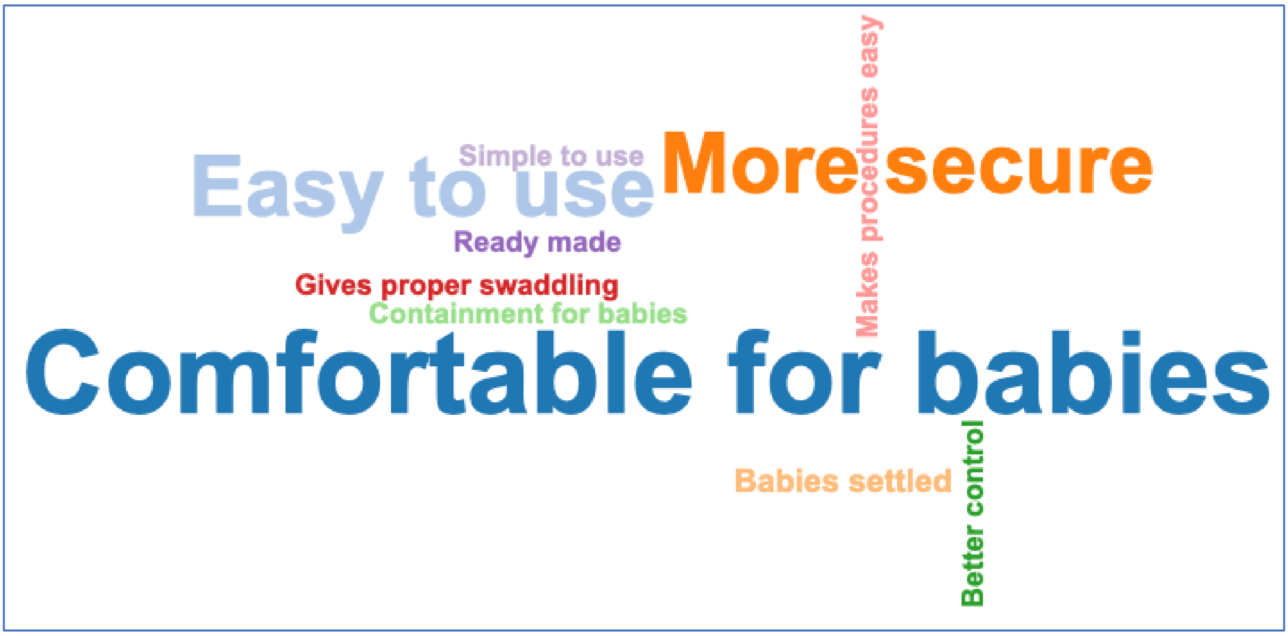

Supplementary Figure 4: Word cloud of reasons given by staff for preference of using the Dandle WRAP over conventional swaddle during ROP screen. The font size represents the frequency of response (the larger the font, the more often the phrase was used by respondents and vice versa).

Supplementary Tables

Supplementary Table 1. Changes in physiology following ROP screening. Changes in physiology in the first 15 min or 12 hours (as indicated) following the start of ROP screens for BIO (Binocular indirect ophthalmoscopy) and UWF imaging. [1]P value indicates whether the ROP screen has a significant effect on physiology compared with zero (see Methods), with both screening types grouped together. [2]P value from the statistical comparison between screening methods. Significance level when correcting for multiple comparisons - α=0.0066. Statistical analysis used linear mixed effects models with infant ID included as a random factor (with random intercept and slope) and demographic characteristics as confounding variables (see Methods). b indicates the coefficient estimate from the model. HR – heart rate measured in beats per minute (bpm), RR – respiratory rate measured in breaths per minute (bpm).

	**All ROP screens**		**BIO**	**UWF imaging**	**Comparison between screening methods**	
	**Mean (±SD)**	**p value[1]**	**Mean (±SD)**	**Mean (±SD)**	**β (95% CI)**	**p value[2]**
**Immediate changes in physiology**						
Maximum HR (bpm)	194.8 ±15.9	<0.0001	199.07 ± 15.97	190.61 ± 14.81	-9.01(-17.64, -0.378)	0.041
Average HR increase above baseline (bpm)	4.30 ±7.26	<0.0001	4.88± 8.72	3.74± 5.52	-0.72 (-4.17, 2.72)	0.68
Minimum oxygen saturation (%)	78.3 ±13.6	<0.0001	79.6.91 ± 14.85	76.92± 12.28	0.43 (-7.20, 8.07)	0.91
Average oxygen saturation decrease below baseline (%)	0.67 ±0.96	<0.0001	0.73 ± 1.08	0.61 ± 0.83	-0.093 (-0.67, 0.48)	0.75
Maximum RR (bpm)	81.7 ± 13.9	<0.0001	79.08 ± 11.96	84.18 ± 15.31	1.16 (-6.81, 9.13)	0.77
Average RR increase above baseline (bpm)	1.95 ±3.20	<0.0001	1.90 ± 2.48	1.99 ± 3.90	1.24 (-0.46,2.94)	0.15
**Physiology in the 12 hours following ROP screen**						
Numbers of tachycardias	1.41 ±2.79	0.81	1.45 ± 3.47	1.39 ± 2.32	-0.09 (-1.65, 1.46)	0.90
Number of bradycardias	1.25 ±2.08	1	0.85 ± 1.50	1.52 ± 2.37	0.42 (-0.76,1.60)	0.47
Number of oxygen desaturations	7.14 ±11.83	0.58	10.55 ± 17.48	4.94 ± 5.18	2.25 (-2.38, 6.89)	0.33
Number of apnoeas	1.71 ±2.72	1	0.95 ± 1.57	2.19 ± 3.19	0.08 (-1.32,1.72)	0.79

Supplementary Table 2: Comparison of physiology changes in infants swaddled with a Dandle WRAP versus controls. Changes in physiology in the 15 minutes or 12 hours (as indicated) following the start of ROP screens. All ROP screens were performed using UWF imaging. Significance level when correcting for multiple comparisons - α=0.0066. Statistical analysis used linear mixed effects models with infant ID included as a random factor and demographic characteristics as confounding variables (see Methods). DW = group swaddled with a Dandle WRAP, HR = heart rate (bpm), RR = respiratory rate (bpm).

	**Control**	**DW**	**Comparison between swaddling methods**	
	**Mean (± SD)**	**Mean (± SD)**	**β (95% CI)**	**p value**
**Immediate changes in physiology**				
Maximum HR (bpm)	193.05 ± 15	188.27 ± 15	-8.05 (-15.8, -0.26)	0.043
Average HR increase above baseline (bpm)	4.08 ± 5.96	3.42 ± 5.18	-3.73 (-7.37, -0.09)	0.045
Minimum oxygen saturation (%)	75.87 ± 10.20	77.76 ± 13.94	5.48 (-3.01,14.0)	0.20
Average oxygen saturation decrease below baseline (%)	0.80 ± 0.99	0.45 ± 0.64	-0.44 (-1.03,0.15)	0.14
Maximum RR (bpm)	85.63 ± 14	82.87 ±16	0.10 (-13.12,13.31)	0.99
Average RR increase above baseline (bpm)	2.12 ± 4	1.88 ±4	-0.93 (-3.43, 1.56)	0.45
**Physiology in the 12 hours following the ROP screen**				
Numbers of tachycardias	1.94 ±2.75	0.71 ± 1.49	-1.84 (-3.73,0.04)	0.054
Number of bradycardias	1.52 ± 2.85	1.43 ± 2.56	0.08 (-1.47, 1.64)	0.91
Number of oxygen desaturations	4.29 ± 4.74	5.71 ± 5.76	1.49 (-2.52, 5.50)	0.45
Number of apnoeas	2.94 ± 3.99	1.29 ± 1.49	-0.65 (-2.76, 1.46)	0.53

Supplementary Table 3: Reasons given by respondents for their choice of ROP screening method. Note, one respondent gave no specific response for having no preference for either the UWF or BIO screening method.

**BIO screen preference**	**UWF imaging screen preference**	**No preference**
"So that we don't have to handle the baby too much. They will be more comfortable."	*"Indirect consumes a lot of time of both health workers and the babies"*	*"Both make baby very upsetting, and uncomfortable. "*
"More comfortable for babies"	*"Babies seem more comfortable "*	*"I haven't seen enough of either to notice a difference or have a preference"*
"In bed procedures can be more hassle free."	*"I think it is better for documentation and being able to get a longer time to look at areas of interest "*	*"I find no matter which procedure they are having the babies don't like being handled no matter how much comfort they are given. "*
"Less stress and handling for the babies "	*"Clinical record for future reference"*	
"Less handling"	*"Quicker"*	
"Less stressed for the baby "	*"Allows for comparison of screening images to help determine if treatment is required. For babies who have had treatment, it allows us to determine if the treatment is working by comparison of finer details. Great tool for teaching. If patients are referred from other units, we can provide optos image so that the local screener can compare our findings (I.e. better communication)"*	
"Can be more comforted and settled "	*"Images can be compared at follow up'*	
"Prevents unnecessary handling and movements "	*"I think it's less painful and baby is less distressed "*	
"It would be more convenient for ventilated babies to do it in the bed itself "		
"Less disturbing for baby"		

Supplementary References

1. Hartley, Moultrie, *et al.* Analgesic efficacy and safety of morphine in the Procedural Pain in Premature Infants (Poppi) study: randomised placebo-controlled trial. *Lancet* **392**, 2595–2605 (2018).

2. Adjei, Purdy, *et al.* New method to measure interbreath intervals in infants for the assessment of apnoea and respiration. *BMJ Open Respir Res* **8**, e001042 (2021).

3. Vergales, Paget-Brown, *et al.* Accurate automated apnea analysis in preterm infants. *Am. J. Perinatol.* **31**, 157–162 (2014).

4. Lee, Rusin, *et al.* A new algorithm for detecting central apnea in neonates. *Physiol. Meas.* **33**, 1–17 (2012).

5. Austin. Balance diagnostics for comparing the distribution of baseline covariates between treatment groups in propensity-score matched samples. *Stat. Med.* **28**, 3083–3107 (2009).

6. The International Classification of Retinopathy of Prematurity Revisited. *Arch. Ophthalmol.* **123**, 991–999 (2005).

7. Chiang, Quinn, *et al.* International Classification of Retinopathy of Prematurity, Third Edition. *Ophthalmology* **128**, e51–e68 (2021).
